# Supplementary material for: Theta-burst microstimulation in the human entorhinal area improves memory specificity
Source: eLife. 2017 Oct 24;6:e29515. doi: 10.7554/eLife.29515 (PMC5655155; doi:10.7554/eLife.29515)
Supplement: Figure 1—source data 1. — Age, gender, handedness, and hemisphere of language dominance of each of the 13 participants (R: right, L: left, A: ambidextrous, B: bilateral, NA: not available (language dominance was not tested)). Antiepileptic drugs refers to medications taken on the day(s) of the experiments. Also shown are the clinically determined seizure-onset zones and stimulated medial temporal lobe (MTL) regions for each subject. Numbers in parentheses indicate the number of individual experimental sessions in which each subject participated. Participants 6 and 13 participated in multiple sessions and received stimulation on each side, but not concurrently. * indicates a stimulated region that fell within the seizure-onset zone for that participant. [file elife-29515-fig1-data1.docx]

| Participant | Age | Gender | Handedness/  Language Dominance | Antiepileptic Drugs | Seizure Onset | Microstimulation Site | |
| --- | --- | --- | --- | --- | --- | --- | --- |
|  |  |  |  |  |  | Left | Right |
| 1 | 20 | M | R/NA | Lamotrigine | Extra-Temporal | Entorhinal White (5) |  |
| 2 | 40 | F | R/L | Lacosamide | Right Medial Temporal |  | Entorhinal White* (4) |
| 3 | 45 | M | L/L | Lamotrigine, Leveltiracetam | Left Medial Temporal | Subiculum* (7) |  |
| 4 | 34 | F | R/L | Lacosamide, Leveltriacetam, Lamotrigine, Oxcarbazepine | Right Medial Temporal | Entorhinal White (4) |  |
| 5 | 35 | F | L/B | Leveltriacetam, Lacosamide | Extra-Temporal | Entorhinal  Gray (2) |  |
| 6 | 30 | M | A/B | Eslicarbazepine Acetate, Lamotrigine, Leveltriacetam | Right Medial Temporal | Entorhinal  Gray (2) | Entorhinal  Gray (1)* |
| 7 | 27 | F | R/NA | Lacosamide, Clobazam | Right Medial Temporal |  | Subiculum* (3) |
| 8 | 20 | M | R/L | Clobazam, Lacosamide | Left Medial Temporal |  | Entorhinal White (2) |
| 9 | 26 | M | R/L | Clobazam, Levetriacetam | Extra-Temporal |  | Entorhinal White (2) |
| 10 | 49 | F | R/L | Lacosamide | Left Medial Temporal |  | Entorhinal White (2) |
| 11 | 35 | F | R/L | None | Left Medial Temporal |  | Entorhinal White (2) |
| 12 | 28 | M | R/NA | Lacosamide | Bilateral temporal |  | Entorhinal White* (1) |
| 13 | 33 | M | R/NA | Lacosamide, Leveltriacetam | Extra-Temporal | Entorhinal White (1) | Entorhinal Gray (2) |
