# Supplementary material for: Theta-burst microstimulation in the human entorhinal area improves memory specificity
Source: eLife. 2017 Oct 24;6:e29515. doi: 10.7554/eLife.29515 (PMC5655155; doi:10.7554/eLife.29515)
Supplement: Figure 3—source data 3. — Each trial’s behavioral outcome was categorized based on whether the viewed portrait was remembered during the test phase or not (total number of trials = 1207 from 13 participants). This metric was modeled as a binary logit response (remembered/missed). Stimulation condition (on/off), stimulation hemisphere (left/right), an interaction term between these two and whether the target image was presented first were specified as factors, which together with normalized trial numbers as a covariate, constituted the model effects. Each factor (row) is quantified by its corresponding (unexponentiated) coefficient (B) and the error associated with it (Std. error and 95% confidence interval), together with whether that term was statistically significant in the model (hypothesis test column). [file elife-29515-fig3-data3.docx]

| Parameter | B | Std. Error | 95% Wald Confidence Interval | | Hypothesis test | | |
| --- | --- | --- | --- | --- | --- | --- | --- |
|  |  |  | Lower | Upper | Wald Chi-Square | df | Sig. (p) |
| Intercept | 1.206 | 0.23 | 0.75 | 1.67 | 26.57 | 1 | 2.54 x 10^-7^ |
| Stim condition x hemisphere | 0.55 | 0.18 | 0.20 | 0.91 | 9.38 | 1 | 0.002 |
| Normalized trial number | -0.21 | 0.16 | -0.52 | 0.10 | 1.72 | 1 | 0.190 |
| Target presented first | -0.33 | 0.17 | -0.67 | 0.004 | 3.74 | 1 | 0.053 |
