# Supplementary material for: Theta-burst microstimulation in the human entorhinal area improves memory specificity
Source: eLife. 2017 Oct 24;6:e29515. doi: 10.7554/eLife.29515 (PMC5655155; doi:10.7554/eLife.29515)
Supplement: Figure 4—source data 1. — Each behavioral metric was modeled as a linear scale response with stimulation hemisphere (left/right), stimulation region (angular bundle/gray matter), and an interaction term between the two (total number of sessions = 40 from 13 participants). The main effects of stimulation hemisphere and stimulation region, but not the interaction, were significant for remembered rate, discrimination index, and target acceptance rate. The interaction term was significant for lure rejection rate. The Wald Chi-Square statistic, degrees of freedom (df), and significance level (p) are presented for each significant effect. [file elife-29515-fig4-data1.docx]

| Behavioral Metric | Effect | Hypothesis Test | | |
| --- | --- | --- | --- | --- |
|  |  | Wald Chi-Square | df | p |
| Remembered Rate | Hemisphere | 14.39 | 1 | 1.49x10^-4^ |
|  | Region | 6.53 | 1 | 0.011 |
| Discrimination Index | Hemisphere | 8.94 | 1 | 2.79x10-^3^ |
|  | Region | 9.12 | 1 | 2.53x10^-3^ |
| Target Acceptance Rate | Hemisphere | 4.47 | 1 | 0.034 |
|  | Region | 17.75 | 1 | 2.52x10^-5^ |
| Lure Rejection Rate | Interaction | 5.615 | 1 | 0.018 |
