# Supplementary material for: Theta-burst microstimulation in the human entorhinal area improves memory specificity
Source: eLife. 2017 Oct 24;6:e29515. doi: 10.7554/eLife.29515 (PMC5655155; doi:10.7554/eLife.29515)
Supplement: Transparent reporting form [file elife-29515-transrepform.docx]

***eLife’s* transparent reporting form**

We encourage authors to provide detailed information *within their submission* to facilitate the interpretation and replication of experiments. If you have any questions, please contact us: [editorial@elifesciences.org](mailto:editorial@elifesciences.org).

**Sample-size estimation**

- You should state whether an appropriate sample size was computed when the study was being designed
- You should state the statistical method of sample size computation and any required assumptions
- If no explicit power analysis was used, you should describe how you decided what sample (replicate) size (number) to use

Please outline where this information can be found within the submission (e.g., page numbers or figure legends), or explain why this information doesn’t apply to your submission:

No explicit sample size computation was performed prior to beginning experiments, however—due to the extreme scarcity of experimental subjects— it is common in the field of invasive human stimulation/physiology to include ~6 subjects, so our initial goal was to have 6 subjects per condition (left/right). We have included 13 subjects, including 6 with left-hemisphere stimulation and 9 with right-hemisphere stimulation (two subjects are counted twice, as they received stimulation on each side).

In addition, many subjects performed the experiment multiple times, resulting in a total of 40 experimental sessions, including 21 sessions with left-sided stimulation and 19 with right-sided stimulation. We used GEEs to analyze our data, because they model the effects of within-subject correlations without losing statistical power (as happens when the average for each subject is computed prior to performing a t-test). Nonetheless, GEEs do not provide a traditional measure of effect size, which in turn limits our ability to perform an *a priori* power analysis for our study.

**Replicates**

- You should report how often each experiment was performed
- You should include a definition of biological versus technical replication
- The data obtained should be provided and sufficient information should be provided to indicate the number of independent biological and/or technical replicates
- If you encountered any outliers, you should describe how these were handled
- Criteria for exclusion/inclusion of data should be clearly stated
- High-throughput sequence data should be uploaded before submission, with a private link for reviewers provided (these are available from both GEO and ArrayExpress)

Please outline where this information can be found within the submission (e.g., page numbers or figure legends), or explain why this information doesn’t apply to your submission:

The experiment was performed 40 times across 13 subjects. This is reported in the main text and methods, and is broken down into sub-categories in the legend of Figure 4. Data is included as Figure 3-source data 1. These 40 experimental sessions resulted in 1207 individual trials (reported in Figure 3 legend and Figure 3-source data 2 legend). This data is available as Figure 3-source data 2.

One subject was excluded from analysis due to severe psychological issues that arose during hospitalization and interfered with her ability to perform tasks. Additionally, trials that received mistimed stimulation in one subject were excluded from analysis. These exclusions are both mentioned in the methods.

**Statistical reporting**

- Statistical analysis methods should be described and justified
- Raw data should be presented in figures whenever informative to do so (typically when N per group is less than 10)
- For each experiment, you should identify the statistical tests used, exact values of N, definitions of center, methods of multiple test correction, and dispersion and precision measures (e.g., mean, median, SD, SEM, confidence intervals; and, for the major substantive results, a measure of effect size (e.g., Pearson's r, Cohen's d)
- Report exact p-values wherever possible alongside the summary statistics and 95% confidence intervals. These should be reported for all key questions and not only when the p-value is less than 0.05.

The primary statistical analysis method employed was Generalized Estimating Equations, described and justified in the main text and the Methods section. For each statistical test, we report exact p-values and 95% confidence intervals. We note that when applying GEEs, we use estimated means and Wald 95% confidence intervals. When we perform a t-test, we show individual data points (Fig 3B) and report Cohen’s-d.

We note at the end of the main text and in the methods that all statistically significant values remain significant under Holm-Bonferroni corrections. Model coefficients are included for Figures 3C and 4A-D in Figure 3-source data 3, and Figure 4-source data 1, respectively.

Please outline where this information can be found within the submission (e.g., page numbers or figure legends), or explain why this information doesn’t apply to your submission:

(For large datasets, or papers with a very large number of statistical tests, you may upload a single table file with tests, Ns, etc., with reference to page numbers in the manuscript.)

**Additional data files (“source data”)**

- We encourage you to upload relevant additional data files, such as numerical data that are represented as a graph in a figure, or as a summary table
- Where provided, these should be in the most useful format, and they can be uploaded as “Source data” files linked to a main figure or table
- Include model definition files including the full list of parameters used
- Include code used for data analysis (e.g., R, MatLab)
- Avoid stating that data files are “available upon request”

Please indicate the figures or tables for which source data files have been provided:

Source data for all session-level analyses is provided as Figure 3-source data 1. This includes data used for Figure 3A-B and Figure 4. Source data for trial-level data is provided as Figure 3-source data 2. SPSS model definitions are included as source code 1-3. Matlab code used for making figures is included as source code 4. Output of the SPSS models hard-coded into the Matlab code, which offers the numerical data that were represented in Figures 3 and 4.
